# Patterned-String Tasks: Relation between Fine Motor Skills and Visual-Spatial Abilities in Parrots

**DOI:** 10.1371/journal.pone.0085499

**Published:** 2013-12-23

**Authors:** Anastasia Krasheninnikova

**Affiliations:** Zoological Institute and Museum, Biozentrum Grindel, University of Hamburg, Hamburg, Germany; UCLA, United States of America

## Abstract

String-pulling and patterned-string tasks are often used to analyse perceptual and cognitive abilities in animals. In addition, the paradigm can be used to test the interrelation between visual-spatial and motor performance. Two Australian parrot species, the galah (*Eolophus roseicapilla*) and the cockatiel (*Nymphicus hollandicus*), forage on the ground, but only the galah uses its feet to manipulate food. I used a set of string pulling and patterned-string tasks to test whether usage of the feet during foraging is a prerequisite for solving the vertical string pulling problem. Indeed, the two species used techniques that clearly differed in the extent of beak-foot coordination but did not differ in terms of their success in solving the string pulling task. However, when the visual-spatial skills of the subjects were tested, the galahs outperformed the cockatiels. This supports the hypothesis that the fine motor skills needed for advanced beak-foot coordination may be interrelated with certain visual-spatial abilities needed for solving patterned-string tasks. This pattern was also found within each of the two species on the individual level: higher motor abilities positively correlated with performance in patterned-string tasks. This is the first evidence of an interrelation between visual-spatial and motor abilities in non-mammalian animals.

## Introduction

The perceptual and cognitive abilities necessary for everyday problem-solving such as foraging vary depending on the ecological niche of a species. For example, estimating distances and spatial relationships between oneself and objects, or between several objects in the environment, requires visual-spatial abilities and is a prerequisite for tracing causal relations among objects. Comparing perceptual and cognitive abilities among species requires a paradigm that allows a broad comparison across species and is easy for a subject to understand and handle [1]. The string-pulling task and its extended versions such as patterned-string tasks fulfil the requirements of being simple and feasible while testing certain abilities such as perceptual capacity [2], means-end knowledge [3], and understanding of spatial relationships [4,5].

A patterned-string task in which the subject must choose between two or more strings, only one of which is connected to a reward, requires both perceptual and cognitive abilities as the subject has to determine the difference in the strings and understand which would lead to the reward. The ability to solve patterned string tasks has been tested in numerous mammals [6-8] and birds [9-11] (in both horizontal and vertical apparatus settings).

Several authors have suggested that fine motor skills play an important role in the ability of a species to solve a string-pulling task [12-14]. According to this sensorimotor argument, usage of feet to manipulate food items and finely tuned beak-foot coordination may both be crucial manipulative skills needed for vertical string-pulling in birds [15]. A large number of different motions performed in a very precise order and involving accurate beak-foot coordination are necessary to pull up and retrieve food attached to the end of a string. Therefore, species that occupy niches which do not require particular sensorimotor skills (for example, a feeding technique which requires fine beak-foot coordination) may be less well equipped for manipulating such objects successfully. The first empirical evidence that finely tuned beak-foot coordination influences success in a vertical string-pulling task came from Magat and Brown [14] who analysed the influence of lateralization on problem-solving. In their study on Australian parrots, all six species that successfully mastered the task use their feet to manipulate food items. The remaining two species, the cockatiels and the budgerigars, which do not use their feet when feeding and do not have pronounced body part coordination, failed entirely in the vertical string-pulling task. It appears that the usage of the feet to manipulate food items is species-specific and could be related to the specific ecological demands faced by a species [14]. Altevogt [16] suggested that fixing an item under the foot or holding it in the foot could be innate. 

A neural basis for an interrelation between visual-spatial and motor skills may be manifested in brain structures. For example, the cerebellum is not, as traditionally assumed, only responsible for motor coordination and motor control, but is also involved in a wide range of processes [17,18]. In humans, both clinical observations [19-21] and functional neuroimaging data [22] showed cerebellar involvement in a variety of visual-spatial tasks. Data from behavioural studies on children underpin the hypothesis of an interrelation between visual processing and fine motor control [23]. In rats, cerebellar lesions provoked impairment in visual-spatial problem-solving and in right /left discrimination [24], and behavioural observations in Kunming mice showed a correlation between non-spatial cognitive and sensorimotor performances [25]. The avian cerebellum shares much histological and physiological similarity with that of mammals [26], including an involvement in visual processing [27]. In large-brained birds, i.e. corvids and parrots, Sultan and Glickstein [28] found enlarged visual and beak-related cerebellar parts, which might be associated with elaborated beak control. Finally, findings from cerebellar lesion study in a songbird suggest that also the avian cerebellum also interrelates motor and cognitive functions [29]. 

Patterned-string tasks have been used to assess a variety of capabilities in animals, but a link to motor-skills has not been tested. The single string task is an appropriate method to test the motor-skills of birds in particular, as the set-up requires complex string manipulations and, presumably, fine beak-foot coordination. Thus, enhanced manipulative skills may facilitate the handling of a vertical string. Patterned-string problems are commonly used to examine the visual-spatial aspects of string pulling [30,31]. If motor and perceptual-cognitive development relies on common mechanisms, a species without pronounced motor skills will probably also lack a predisposition to perform certain aspects of patterned-string problems. 

Here, I examine the string-pulling performance of two Australian parrot species which show differences in feeding technique and hence in fine beak-foot coordination, the galah (*Eolophus roseicapilla*) and the cockatiel (*Nymphycus hollandicus*). Both species are widely distributed on the Australian continent. They share a preference for open, semi-arid habitats close to water [32,33], and thus occur partially sympatrically. Both species subsist primarily on small seeds from native or cultivated plants and grasses [14,34,35], and both forage on the ground, but only one of them, the galah, uses its feet to manipulate food items. Hence, as they share various ecological parameters, such as diet and feeding mode but differ in their manipulative capabilities, these two species present an interesting opportunity to test the hypothesis that certain motor skills need to be present to perform well in patterned-string tasks requiring specific visual-spatial skills (e.g. distance perception, and visual-spatial processing). By using string-pulling and patterned-string tasks with different degrees of difficulty, I test (1) motor skills, and (2) visual-spatial abilities in both species. I hypothesized that the galahs would outperform the cockatiels in the motor task due to their pronounced beak-foot-coordination. Assuming an interrelation between visual-spatial skills (e.g. estimating distances and spatial relationships between objects) and motor performance I also hypothesized that the galahs would solve the patterned-string tasks more successfully than the cockatiels. 

## Results

### Comparison between Species

**Figure 1:**
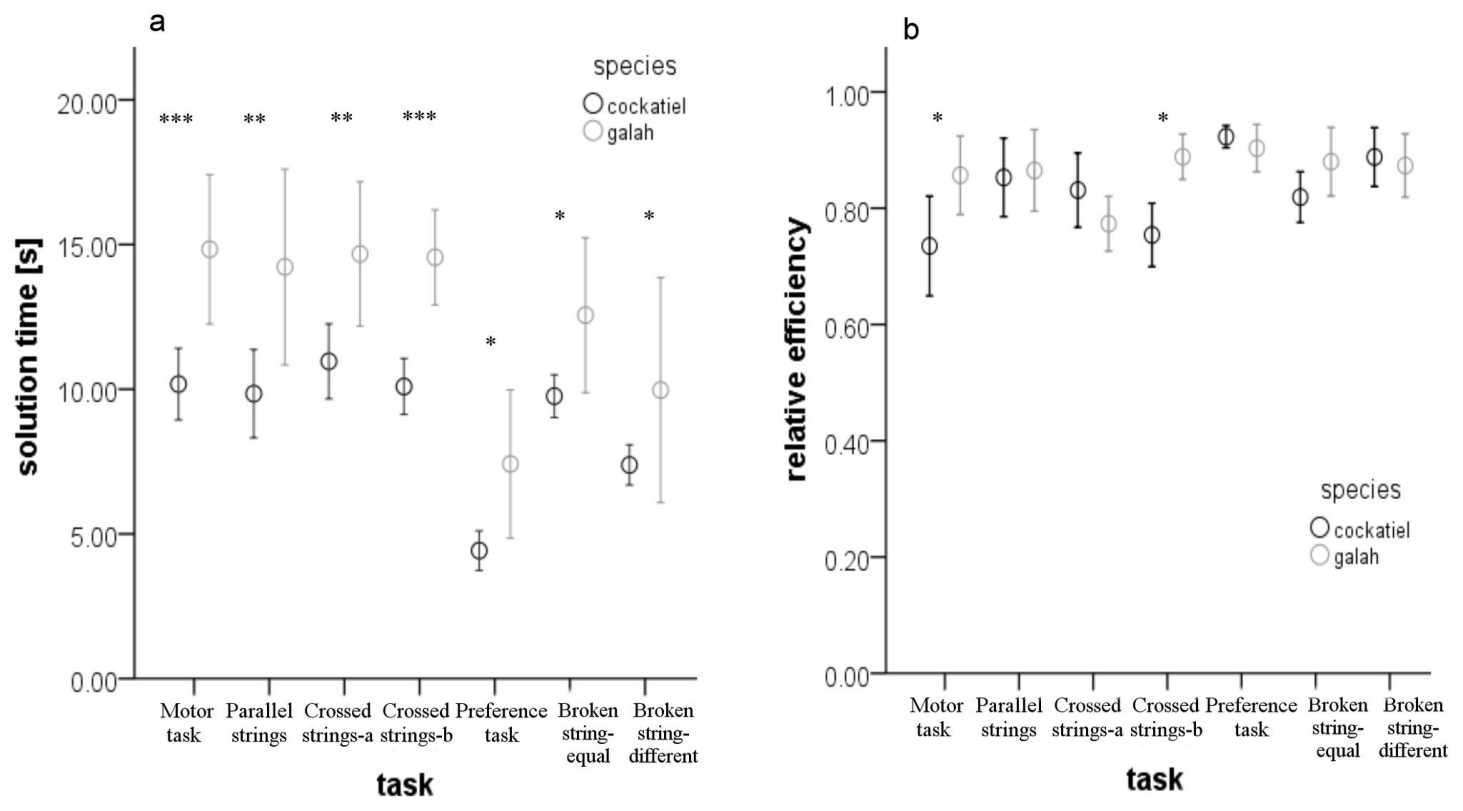
Performance across tasks. The time (a) needed to pull the rewarded string and the relative efficiency (b) of string-pulling shown in the motor task and in the patterned-string tasks. The circles represent the mean values and the whiskers represent the standard errors. The stars indicate the tasks where the differences between the species were significant; ^*^ P<0.05, ^**^ P<0.01, ^***^ P<0.001. Relative efficiency was calculated by the formula: (frequency of effective actions – frequency of ineffective actions)/total number of actions.

In the motor task (T1), individuals of both species performed very well and pulled the rewarded string spontaneously. All but one galah and one cockatiel pulled the string on their first attempt. Although the relative length of the string was the same for each species (twice as long as the body size), the galahs needed significantly longer to pull it (GLMM, factor “species”: Chi^2^ = 14.189, *df* = 1, *P* < 0.0001), but showed a greater relative efficiency in their string-pulling behaviour than the cockatiels (GLMM, factor “species”: Chi^2^ = 4.9698, *df* = 1, *P* = 0.026). The number of pulls needed to reach the reward differed across individuals, varying in both species between 3 and 7 pulls. However, in patterned-strings tasks (T2-T7) the relative efficiency did not differ significantly (with the exception of the crossed strings-b task, T4), despite significant differences in time (Figure 1). 

### Techniques used

**Figure 2:**
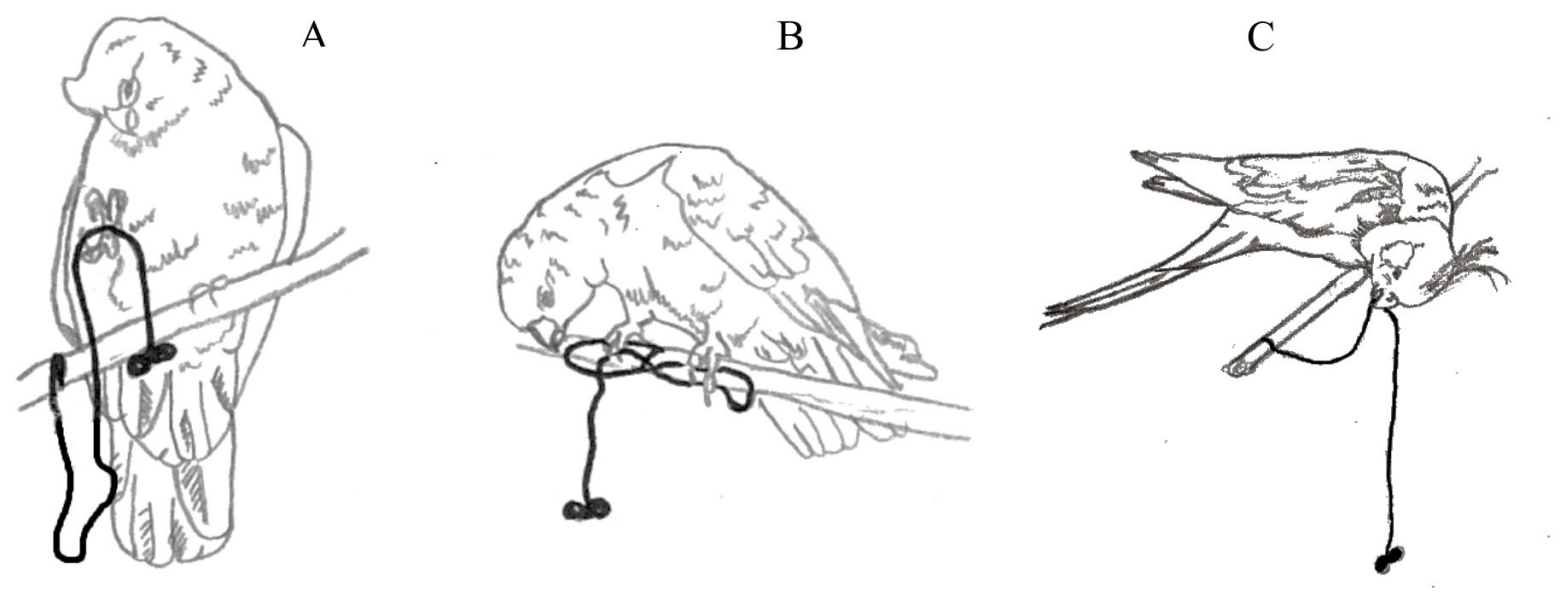
Different techniques used. Three samples for strategies used by birds to obtain the reward (A – upright pulling, occurred only in galahs; B – looping, occurred in galahs and cockatiels, C – sliding, occurred only in cockatiels) .

Although the task appears to lend itself to straightforward solutions, considerable variation in techniques and in the frequency with which they were used were displayed and appeared both across and within species. Generally, the galahs manipulated the string with the foot rather than only stepping on it to fix it to the perch, whereas the cockatiels used the foot only to step onto the looped string (Figure 2). Overall, the group of galahs employed five and the group of cockatiels four different techniques when confronted with the various tests. Some subjects used elements of two different techniques to pull the string. Upright pulling occurred in galahs only, whereas sliding was shown only by cockatiels. Two methods, looping and side walking were shown by all subjects. In both species, there was considerable intraspecific variation in the preference for the techniques used (one-way ANOVA, F = 6.58, df = 5, P = 0.009, F = 3.04, df = 9, P = 0.03, respectively). There was also a significant difference in the mean BFC score (one-way ANOVA, F = 10.95, df = 1, P = 0.01) reflecting the fact that on average galahs used techniques with a higher BFC score. 

### Patterned string tasks

**Figure 3:**
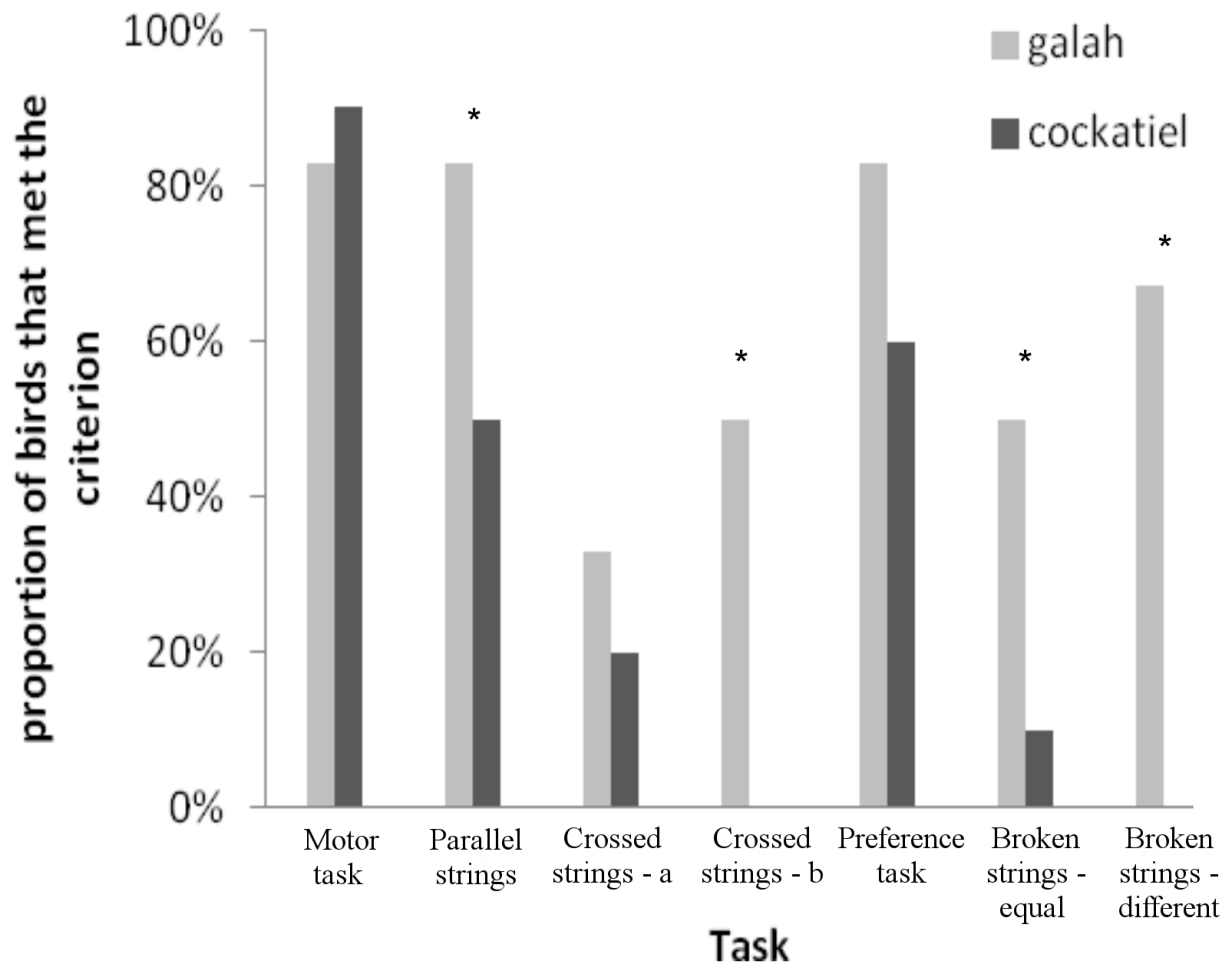
Proportion of birds that met the success criterion. The success criterion means choosing the correct string in the first trial and in at least 8 out of 10 trials in total. The values for the preference task (T5) show the preference for a shorter string when presented with two rewarded strings of different length. The stars indicate the tasks where the differences between the species were significant; ^*^ P<0.05.

The galahs scored significantly higher than the cockatiels in patterned string tasks (T2-T7): they had a higher number of successfully solved patterned-string tasks (GLMM with individual as random factor, Chi^2^ = 5.341*, df* = 1*, P* = 0.019), i.e. in the number of tasks where they made the right choice the first time and rarely made any errors thereafter. The proportion of birds that met the success criterion also differed between species (Figure 3), being on average significantly higher for galahs than for cockatiels (GLMM with task as random factor, Chi^2^ = 7.756, *df* = 1, *P* = 0.005). In task 4 and task 7, only some galahs (50 % and 75 %, respectively) met the criterion. Success varied between species depending on the task (GLMM, species*task, Chi^2^ = 3.712*, df* = 1*, P* = 0.034). The individual performance in the patterned-string tasks is summarised in Table 1.

**Table 1:** Individual performance showing how many trials the subject solved successfully and the VSA scores of the subjects tested.

Subject		Task						VSA score
		T2	T3	T4	T5	T6	T7	
Galah	G1m	**8**	9	**9**	**8**	**8**	**10**	5
	G2f	5	**8**	5	**10**	**8**	**8**	4
	G3f	**8**	7	**8**	**8**	8	**8**	4
	G4m	**8**	6	6	**9**	5	4	2
	G5f	**8**	8	6	**8**	6	4	2
	G6m	**9**	**8**	**8**	5	**9**	**9**	5
Cockatiel	C1m	**9**	6	6	**10**	8	5	2
	C2m	**10**	9	8	6	**8**	6	2
	C3m	4	6	5	**9**	5	4	1
	C4m	5	**8**	3	6	4	3	1
	C5m	6	6	8	**8**	5	5	1
	C6f	**8**	5	3	**8**	4	2	2
	C7f	6	4	4	5	4	1	0
	C8f	6	6	5	8	5	2	0
	C9m	**8**	5	4	**10**	8	6	2
	C10m	**9**	**8**	5	**10**	3	3	3

Numbers represent the number of correctly solved trials (out of 10 in total) per task; the numbers in bold show that the criterion (at least 8 correct choices out of 10 trials) was met; VSA score refers to the number of patterned-string tasks (T2-T7) in which the subject met the criterion; m=male, f=female.

### Interaction between Motor Skills and Perceptual Skills at the Individual Level

**Figure 4:**
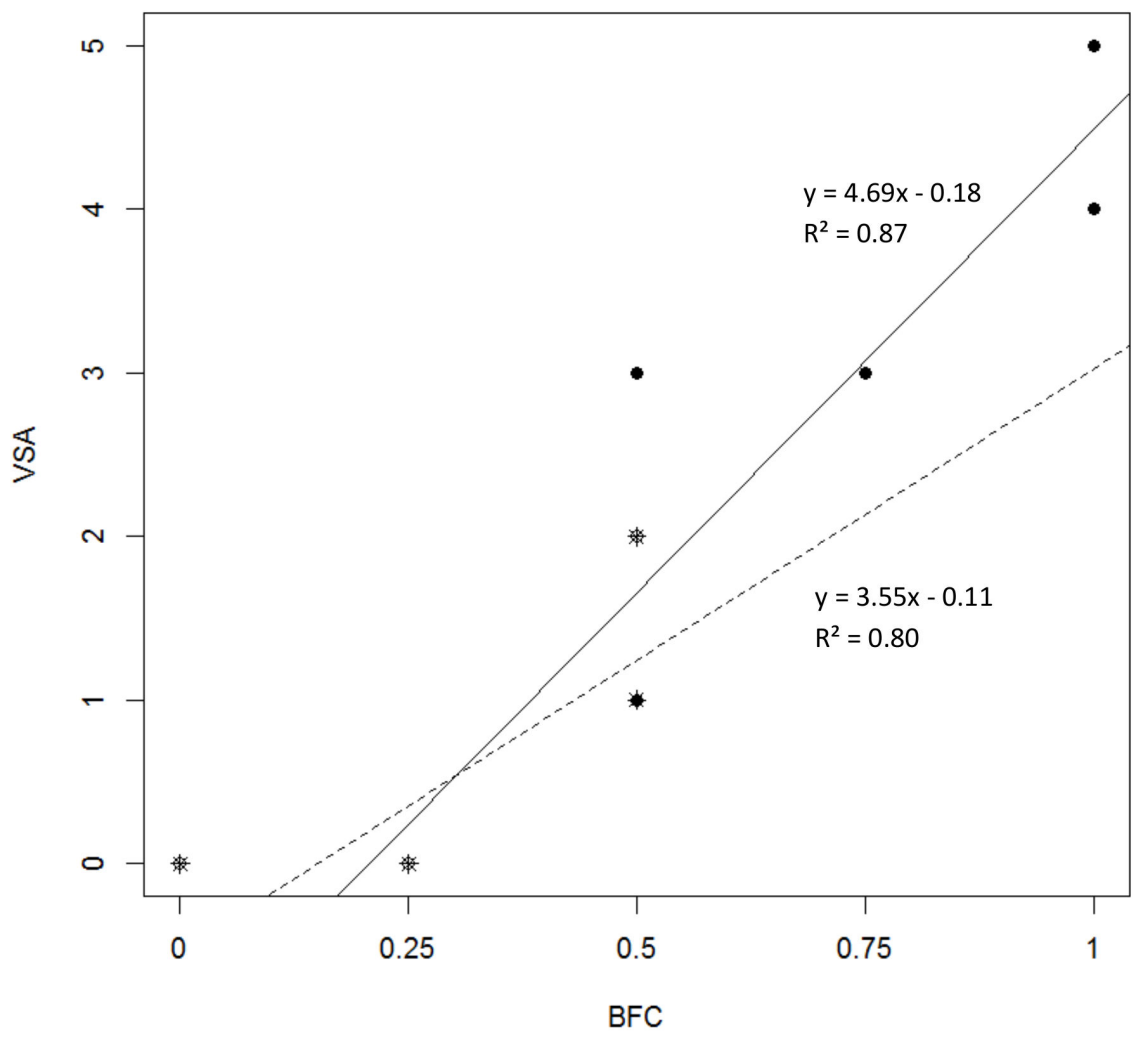
Correlation between the visual-spatial skills and the motor skills. Spearman rank correlation using the score for beak-foot-coordination (BFC score), measured in terms of the extent of foot usage in the technique preferred when solving the novel motor task (T1), and the score for visual-spatial abilities (VSA score) which reflects the number of correctly solved patterned-string tasks (T2, T3, T4, T5, T6, and T7). Filled circles represent the data for the galahs and the stars represent the cockatiel data; the dashed line represent regression line (*F* = 36.14, *P* = 0.003) for the galah data and the solid line (*F* = 9.46, *P* = 0.008) the cockatiel data. The score for the preferred technique refers to the technique used in over 70 % of all trials in T1. If no technique was clearly preferred (i.e. above 70 % threshold), the mean score for the two most frequently used techniques was calculated.

At the individual level, preferences for different solving techniques were found. Several individuals switched techniques between trials, but no consistent pattern was detectable. In both species, a correlation between motor skills in terms of the extent of beak-foot coordination and the overall performance in patterned-string tasks (number of meeting the criterion) was found (Spearman rank correlation; *r*_*s*_ = 0.94, *P* = 0.005 for galahs, and *r*_*s*_ = 0.79, *P* = 0.005 for cockatiels). The higher the score for beak-foot coordination measured in T1, the higher was the number of successfully solved patterned-string tasks (Figure 4). No correlation was found between BFC and time or between BFC and relative efficiency (see data in Table S1). 

## Discussion

Contrary to expectation, the cockatiels managed the vertical string-pulling problem despite the fact that they do not naturally perform pronounced beak-foot coordination activities. Most individuals of both species pulled the single baited string spontaneously. Hence, I found no support for the hypothesis that the use of feet and beak in the feeding context indicates the presence of the manipulative skills needed for successful string-pulling. However, members of the two species used different sets of techniques to pull the string and the techniques clearly differed in terms of the extent of beak-foot coordination shown. Whereas the galahs manipulated the string with their feet (grabbed it to pull it through the beak in alternate sequences), the cockatiels either just lifted the string by drawing it up through their beak without using their feet at all or they used a foot but only to step on the looped string and to fix it to the perch. Therefore, the performance of the cockatiels showed that using the feet while feeding is not necessary to solve the vertical string-pulling problem, but that it may well determine how the task is solved. 

A larger difference between the species was found in the tasks that tested visual-spatial skills (T2, T3, T4, T6, and T7). Measured in terms of how many trials were solved correctly and of how many individuals were successful at a specific task, the galahs outperformed the cockatiels. Three galahs and one cockatiel were able to visually determine a physical connection between objects (T6), whereas only three galahs (and no cockatiel) were able to distinguish between two crossed strings of the same colour (T4). Most of the subjects in both species preferred the shorter rewarded string (T5), thus choosing the most efficient solution. However, only galahs were able to suppress this preference when the shorter string was not connected to a reward (T7), thus showing some kind of understanding that the string must be connected to the reward to work properly. Note that both species pulled the string in patterned string tasks, each using techniques reflecting the extent of their motor skills – in this way both species had the skills to solve the patterned testing problem – but the capacity to recognize the spatial relation between string and reward appeared to differ between them. Of course, birds can fail for other reasons, such as motivation [3]. However, as the birds participated in the test, but did not meet the criterion, motivation was probably not a key factor. 

Clearly, two species are not sufficient to draw conclusions about an interrelation between motor and specific visual-spatial skills needed to recognize spatial relations between objects in parrots. However, as this is the first attempt to investigate the possibility of such an interrelation, the patterns are encouraging and call for more research. Support can be found in a within-species comparison, as fine beak-foot coordination and performance in patterned-string tasks were positively correlated. The higher the score for motor abilities (defined as a preference for techniques that require finer beak-foot coordination) was, the more successful were the performances achieved by the subject. Furthermore, the published literature on vertical patterned string problems in parrots shows that all species tested so far (keas; [39], hyacinth macaws, Lear’s macaws, and blue-fronted amazons; [9]) used techniques with pronounced beak-foot-coordination, performing similarly at the patterned string tasks to the galahs in the present study. Furthermore, spectacled parrotlets (*Forpus conspicillatus*) that do not use their feet to manipulate food items were found to use techniques with a medium beak-foot coordination score when pulling a single rewarded string [40]. However, when presented with a set of patterned-string tasks spectacled parrotlets performed unexpectedly well [41]. These findings suggest that there might be a constellation of reasons for success or failure in these types of tests, i.e. there could be further mechanisms responsible for differences in ability to solve patterned-string tasks. However, further studies are needed to determine these possible reasons.

Contrary to the findings of Magat and Brown [14], who suggested that species that failed to pull the string probably never encountered problems requiring advanced manipulative skills, the present study provides evidence that prior fine motor skills such as pronounced beak-foot coordination are not necessary for the ability to perform string-pulling in general. Interestingly, while findings in the present study suggest that fine motor coordination may be interrelated with visual-spatial skills, it appears that success in other tasks such as object permanence may not be. In the study comparing object permanence in four parrot species [42], a cockatiel and a budgerigar, species which do not hold their food with the foot, exhibited object permanence just as well as a grey parrot and an Illiger’s macaw – both “feet users” – did. 

The patterned string task has been suggested to provide a reasonable simulation of natural foraging situations encountered by frugivore species [43]. For example, common marmosets have been observed to pull branches of trees towards them that hold fruit but that are too small to walk across [44,45]. As the vegetation of trees is often dense, it is likely that the marmosets have to choose the right branch to pull. The same foraging pattern is true for parrot species feeding on fruits and plants [46-48]. However, most of the diet of both galahs and cockatiels consists of seeds gathered mainly on the ground [34,35]. The differences found between the two species are thus particularly remarkable, as it is unlikely that the tasks used in this study favour the ecological niche of one species more than that of the other. Furthermore, parrots’ exploratory play and their climbing mode of locomotion require strong visually guided beak usage to manipulate and explore external objects. Indeed, parrots show an enlargement of specific visual and beak-related cerebellar parts, suggesting that this may be related to their repertoire of visually guided goal-directed beak behaviour [49]. 

A potential limitation of the present study may be that when testing subjects jointly, different social learning speeds may have influenced the group performance as a whole as well as individual performance. If the birds had used social learning, a sequential pattern of similarity in the techniques applied could be expected. Yet, I found no such pattern: the birds that followed after the first one used different techniques, suggesting that imitation did not play a role. However, types of social learning such as local enhancement, object enhancement or social facilitation – that only guide the attention of the observer to a location or item but still require individual learning by trial and error – likely played a motivational role in galahs. All six birds in the group showed an interest in the tasks, while only 10 of 22 cockatiels participated in the tests even though all subjects were able to observe the successful individuals, and even though monopolization of the set-up was prevented by the presence of several apparatuses. Finally, birds that initially failed a task did not improve their performance in the following trials even though they clearly observed successful companions. Therefore, social learning did not appear to influence the birds’ success rate. 

Further studies using standardized paradigms to test visual-spatial and motor skills across a wide range of parrot species are necessary to show whether the patterns found at the individual level are consistent across a wider range of species and to support the possibility that specific abilities such as visual-spatial skills may interrelate with motor skills not only in mammals but also in birds. 

## Materials and Methods

### Study Subjects

Six galahs and ten cockatiels were tested. All parrots were hatched in a zoo and were raised by their parents. No artificial toys were available, but green branches were provided regularly for playing and nibbling. 

The galahs were kept in a walk-through outdoor aviary (12 x 7 x 5 m) with an adjacent indoor aviary (6 x 1.6 x 2.5 m) at Tierpark Gettorf, Germany. The group contained five adults and one subadult (3 males, 3 females), which were not related to each other. All individuals participated in the study. Birds were fed every day between 9 a.m. and 11 a.m. with parrot pellets and fruits. The indoor aviary was lit by several windows and provided with several perches and a nestbox. The outdoor aviary contained several trees and a trunk. Water was available *ad libitum* and vitamins were given twice a week. The galahs were housed together with a group of golden pheasants (*Chrysolophus pictus*). The zoo visitors were able to enter the outdoor aviary and to feed the animals with zwieback. 

The cockatiels were kept together with budgerigars (*Melopsittacus undulatus*) in a walk-through outdoor aviary (18 x 5 x 7 m) with an adjacent indoor aviary (11 x 1.6 x 2.5 m) at Tierpark Gettorf, Germany. The group contained 20 cockatiels (15 adults, 5 juveniles), and over 60 budgerigars. Birds were fed every day between 9 a.m. and 11 a.m. with a mixture of different fruits and seeds. Water was available *ad libitum* and vitamins were given twice a week. The indoor aviary was lit by several windows and provided with several perches and nestboxes. The outdoor aviary contained several trees, branches and trunks. The zoo visitors were able to enter the outdoor aviary and to feed the animals with proso millet (*Panicum miliaceum*). Ten cockatiels showed no interest and did not approach the string-pulling apparatus: thus, they were excluded from the analyses. The size (thickness) of the string allowed the budgerigars to land on the string; they did not show any pulling attempts. Therefore, the budgerigars’ performance was not included in the analyses. 

The galah and cockatiel aviaries were close to each other, so that the birds were not acoustically isolated, but a barrier prevented any visual cues from one group to the other during the experiment in the outdoor aviary. All subjects of each species could be individually recognized at all times during the experimental sessions.

No subject had contact with string-like objects or had been trained in any object-pulling task prior to the present experiments. The animal care during the study was performed by the regular zoo keepers. The daily feeding conditions were adapted to the testing situation. The experiments reported were integrated into the daily routine as part of the regular animal welfare activities. After the study, all tested birds remained in their respective flocks. 

### Experimental set-up

The birds were given their regular variety of seeds on test days, but they were deprived of their preferred fruits and vegetables on those days. Water was available *ad libitum*. To keep birds motivated, highly favoured food rewards were used which were not available outside the experimental context: peanut halves for the galahs and pieces of foxtail millet (*Setaria italica*) for the cockatiels. To reduce any potential neophobic reaction towards the strings, two days prior to the beginning of the experiments small pieces of string (<5 cm) were left hanging on the lateral wire walls of the aviaries. The birds had access to the string, but could not pull it or remove it from the wire. Each subject was presented with 10 trials per task. I conducted two sessions per day, one in the morning (from 9 a.m.) and one in the afternoon (from 3 p.m.). Tests were presented in the same order for both species. To ensure that the bird’s performance in patterned-string tasks was not based on local enhancement, that is, choosing the string that had been manipulated last or that had moved last, I always manipulated both strings. To minimize the possibility of monopolization of the set-up, several apparatuses were presented. Trials ended when a subject reached the free end of the string (regardless of whether it had the reward attached to it or not), or after a pre-determined maximum of 5 min, whichever came first. In all choice tasks, the colours and sides associated with the reward attached to the string were alternated randomly across trials. The weight of both the string and the reward was appropriately adjusted for each species. The distance between the strings was twice the body length of the target species. To cross the strings in the crossed string configuration of patterned-string tasks, I used thin wire attached to lateral walls or poles and visible for the birds. The string that every bird first interacted with was scored as its choice in every trial. The choice was scored as ‘correct’ if the subject started with a pulling action on the rewarded string and reached the end of the string. All tests were video recorded. The solution time, i.e. time needed to reach the food, the number of efficient (“pulls”) and inefficient (“drops”) actions, and the techniques used to pull the string were noted for subsequent analysis of the birds’ behaviour.

The subjects were tested jointly in their respective groups to simulate conditions in which subjects deal with a novel problem (e.g. new food sources) in the natural environment, where usually a set of individuals is faced with a new situation at the same time. 

#### String-pulling task to assess body part coordination as a measure of motor ability

Motor task (T1): This task tested the parrots’ ability to pull up a reward suspended from a horizontal perch by a single string and examined the techniques used to obtain this reward. 

#### Patterned-string tasks to assess visual-spatial skills as a measure of perceptual ability

Parallel strings (T2): To test if string-pulling behaviour is food-directed, two strings, one with the reward attached as before and one without, were simultaneously presented to the birds. Pulling up the string with the reward more frequently than expected by chance would indicate that the subject could recognize the string as a means to obtain the reward even if string-pulling behaviour in T1 had been self-rewarding. 

Crossed strings – a (T3): To assess whether the parrots’ choice was based on the spatial or the functional relationship between string and reward, I crossed the strings. If their choice was based on the functional connection between food and string, they would pull the baited string. If the choice was based on the spatial relationship only, they would pull the string directly above the bait, as in the earlier trials. In T3, two differently coloured strings (green/white or green/yellow or white/red) were used to allow the birds to trace the strings from one end to the other more easily, assuming that both species have similar colour sensitivity [36]. Thus, the birds could either visually trace the paths signalled by the strings (which is easier to discern when the strings are differently coloured) or choose the string with the same colour as that connected to the reward (which means that they were at least able to recognize the connection principle). The rewarded strings, and therefore the rewarded colours, were varied randomly across trials, so that any association rule of a particular colour with the food was excluded (e.g. choosing the colour that has been last rewarded would lead to a failure at the task). 

Crossed strings – b (T4): This was in principle the same test as in T3, but with two crossed strings of the same colour (white/white or red/red or green/green), and thus expected to be more difficult for the birds to discern.

Preference task (T5): To test if the subjects show a preference for the shorter string (with a reward which could be obtained more easily), two rewarded strings of different lengths were presented.

Broken strings – equal (T6): To test the ability to visually determine whether or not objects are physically connected, two strings of equal length were presented to the subjects. While one string was connected to a reward, the other one had a gap between string and reward. Both rewards were placed on a small platform on a wire (attached to the lateral walls or poles). The distance between the string and the unconnected reward was 5 cm.

Broken strings – different (T7): To test if the birds realize that the string must be connected to the reward in order to work properly, I presented two strings of different length as in T5, but the shorter string was disconnected from the reward. To succeed the birds would abandon any preference for the short string, and chose the longer, rewarded string instead. 

The position of the rewarded string in choice tasks was determined randomly across the sessions by tossing a coin.

### Analysis

For each species, I calculated the proportion of birds that met the criterion of choosing correctly in the first trial and in at least 8 out of 10 trials in total. For the analysis of quantitative differences between species, I performed a generalised linear mixed model (GLMM) analysis using lmers (package ‘lme4’, [37]) in R 2.15.2 [38], with ‘individual’ as random factor to assess the difference in the proportion of successfully solved patterned-string tasks, and with ‘task’ as random factor for differences in the proportion of the birds that met the success criterion. The distribution was set as binomial for event data (success or no success) with logit link function and Gaussian (identity link function) for continuous variables (e.g. time, relative efficiency).

Each individual received a score for its relative efficiency in solving the task by comparing frequencies of effective reactions, namely “pulls”, and ineffective reactions, namely “drops”. The score was calculated using the formula: (frequency of effective actions – frequency of ineffective actions) / total number of actions (see also [9]). 

To quantify the extent of beak-foot-coordination (BFC), a score was calculated quantifying the extent of foot usage in the technique preferred when solving the novel motor task (T1). A score of 0.5 was assigned to pulling the string with the beak and using the foot just to fix the string on the perch: this was considered moderate coordination. Conversely, pulling the string first with the bill and then using the foot to pull the rest of the string while holding it in the bill and repeating the foot movements (i.e. to the bill to hold the string in the foot and away from the bill with the string in the foot to gain more string, repeating this action up to seven times) was considered highly coordinated and scored as 1.0 (being in general the same movement as the touching of the nose used as a part of the LOS test measuring fine motor skills in children [23]; techniques where the foot was not used at all scored 0 (Table 2). The primary technique used was defined as the one used for more than 75 % of the total number of trials. For example, when the subject used the foot just to fix the string to the perch in over 75 % of its trials, its overall beak-foot-coordination was scored as 0.5. 

**Table 2:** Definition of the techniques used by the subjects to obtain the reward.

Technique	Definition	BFC score
Sliding	pulling up the string through the bill without fixing or holding it with the foot	0
Flip	reaching down and flipping the string to the other side of the perch	0
Looping	reaching down, pulling up string with the beak, placing the foot on the string, letting go of the string with the beak, remaining in place, reaching down again	0.5
Side walking	reaching down, pulling up the string with the beak, walking to the side of the perch, placing the foot on the string, and reaching down again	0.5
Turn	turning the whole body 180° while holding the string and stepping on the additional string with the feet	0.5
Upright pull	pulling up the string till the body is in a completely upright position, holding with the beak, and gaining more string by grabbing it with the foot	1

BFC score is the beak-foot coordination coefficient.

To quantify the performance in patterned-string tasks, a score for visual-spatial abilities (VSA) was calculated using the number of patterned-string tasks (T2, T3, T4, T5, T6, and T7) in which the subject met the success criterion – i.e. reaching the reward in at least 8 out of 10 trials.

Finally, Spearman rank correlations were computed between motor and visual-spatial skills using both scores, to assess the interaction between motor and visual-spatial skills within both species. 

### Ethical Notes

All data collection was carried out in accordance with the guidelines of the University of Hamburg and with permission of the Tierpark Gettorf, Germany. The present study was strictly non-invasive and based on behavioural observations; all reported experiments were classified as non-animal experiments and required no approval from the relevant body in accordance with the German Animal Welfare Act (Federal Law Gazette I, p. 1094, Section V, Article 7).

## Supporting Information

Table S1 **Individual data for solution time, relative efficiency, and BFC.** Spearman's rank correlation coefficients and significance level are given for intraspecific correlations between BFC and solution time and between BFC and relative efficiency in each of the task.
(DOCX)
