# Supplementary material for: Patterned-String Tasks: Relation between Fine Motor Skills and Visual-Spatial Abilities in Parrots
Source: PLoS One. 2013 Dec 23;8(12):e85499. doi: 10.1371/journal.pone.0085499 (PMC3871688; doi:10.1371/journal.pone.0085499)
Supplement: Table S1 — Individual data for solution time, relative efficiency, and BFC. Spearman's rank correlation coefficients and significance level are given for intraspecific correlations between BFC and solution time and between BFC and relative efficiency in each of the task. (DOCX) [file pone.0085499.s001.docx]

| Species | ID | Solution time $\overline{x}$ ± SD | Relative Efficiency | BFC | Correlation BFC-solution time | | | Correlation BFC-relative efficiency | |
| --- | --- | --- | --- | --- | --- | --- | --- | --- | --- |
|  |  |  |  |  | r_s_ | p | | r_s_ | p |
| Task 1 | | | | | | | | | |
| galah | G1m | 14.62 ± 5.1 | 0.73 | 1.00 | 0.43 | 0.397 | | -0.51 | 0.295 |
|  | G2f | 14.17 ± 4.39 | 0.89 | 0.50 |  |  |  |  |  |
|  | G3f | 18.17 ± 4.01 | 0.88 | 0.75 |  |  |  |  |  |
|  | G4m | 10.70 ± 5.13 | 0.90 | 0.50 |  |  |  |  |  |
|  | G5f | 15.37 ± 4.19 | 0.85 | 0.50 |  |  |  |  |  |
|  | G6m | 16.00 ± 4.84 | 0.89 | 1.00 |  |  |  |  |  |
| cockatiel | C1m | 10.47 ± 3.19 | 0.87 | 0.50 | 0.24 | 0.505 | | 0.62 | 0.054 |
|  | C2m | 7.94 ± 2.32 | 0.77 | 0.50 |  |  |  |  |  |
|  | C3m | 11.48 ± 3.39 | 0.47 | 0.25 |  |  |  |  |  |
|  | C4m | 8.12 ± 1.42 | 0.76 | 0.50 |  |  |  |  |  |
|  | C5m | 7.63 ± 1.34 | 0.71 | 0.00 |  |  |  |  |  |
|  | C6f | 11.37 ± 2.69 | 0.81 | 0.50 |  |  |  |  |  |
|  | C7f | 11.87 ± 2.17 | 0.63 | 0.25 |  |  |  |  |  |
|  | C8f | 9.64 ± 2.41 | 0.68 | 0.25 |  |  |  |  |  |
|  | C9m | 12.20 ± 1.96 | 0.86 | 0.50 |  |  |  |  |  |
|  | C10m | 11.05 ± 2.17 | 0.79 | 0.50 |  |  |  |  |  |
| Task 2 | | | | | | | | | |
| galah | G1m | 9.83 ± 3.09 | 0.86 | 1.00 | -0.31 | 0.542 | 0.29 | | 0.566 |
|  | G2f | 12.84 ± 3.12 | 0.92 | 1.00 |  |  |  |  |  |
|  | G3f | 18.37 ± 6.06 | 0.96 | 0.75 |  |  |  |  |  |
|  | G4m | 14.77 ± 3.96 | 0.84 | 0.75 |  |  |  |  |  |
|  | G5f | 12.27 ± 2.67 | 0.84 | 0.50 |  |  |  |  |  |
|  | G6m | 17.29 ± 11.30 | 0.77 | 0.75 |  |  |  |  |  |
| cockatiel | C1m | 7.70 ± 1.91 | 0.87 | 0.50 | 0.25 | 0.484 | 0.44 | | 0.198 |
|  | C2m | 7.60 ± 3.00 | 0.95 | 0.50 |  |  |  |  |  |
|  | C3m | 7.44 ± 5.25 | 0.75 | 0.00 |  |  |  |  |  |
|  | C4m | 8.96 ± 1.21 | 0.96 | 0.50 |  |  |  |  |  |
|  | C5m | 8.25 ± 1.40 | 0.91 | 0.00 |  |  |  |  |  |
|  | C6f | 9.92 ± 3.09 | 0.89 | 0.50 |  |  |  |  |  |
|  | C7f | 12.23 ± 3.12 | 0.77 | 0.25 |  |  |  |  |  |
|  | C8f | 12.21 ± 2.46 | 0.95 | 0.50 |  |  |  |  |  |
|  | C9m | 12.75 ± 2.61 | 0.77 | 0.50 |  |  |  |  |  |
|  | C10m | 11.41 ± 2.11 | 0.71 | 0.25 |  |  |  |  |  |
| Task 3 | | | | | | | | | |
| galah | G1m | 12.27 ± 7.08 | 0.73 | 0.75 | 0.52 | 0.287 | 0.63 | | 0.178 |
|  | G2f | 13.57 ± 3.69 | 0.77 | 0.75 |  |  |  |  |  |
|  | G3f | 16.75 ± 2.83 | 0.82 | 0.75 |  |  |  |  |  |
|  | G4m | 12.79 ± 3.80 | 0.81 | 0.75 |  |  |  |  |  |
|  | G5f | 14.36 ± 3.04 | 0.71 | 0.50 |  |  |  |  |  |
|  | G6m | 18.31 ± 7.03 | 0.80 | 1.00 |  |  |  |  |  |
| cockatiel | C1m | 10.29 ± 2.25 | 0.90 | 0.50 | -0.04 | 0.907 | 0.55 | | 0.100 |
|  | C2m | 9.84 ± 1.99 | 0.89 | 0.50 |  |  |  |  |  |
|  | C3m | 13.96 ± 10.46 | 0.90 | 0.50 |  |  |  |  |  |
|  | C4m | 9.37 ± 1.58 | 0.89 | 0.50 |  |  |  |  |  |
|  | C5m | 8.20 ± 1.99 | 0.89 | 0.50 |  |  |  |  |  |
|  | C6f | 11.31 ± 2.02 | 0.82 | 0.25 |  |  |  |  |  |
|  | C7f | 12.52 ± 2.04 | 0.75 | 0.50 |  |  |  |  |  |
|  | C8f | 9.45 ± 1.57 | 0.84 | 0.25 |  |  |  |  |  |
|  | C9m | 12.46 ± 1.70 | 0.62 | 0.25 |  |  |  |  |  |
|  | C10m | 12.22 ± 1.58 | 0.81 | 0.50 |  |  |  |  |  |
| Task 4 | | | | | | | | | |
| galah | G1m | 14.20 ± 5.71 | 0.86 | 1.00 | 0.39 | 0.441 | -0.67 | | 0.148 |
|  | G2f | 13.84 ± 3.20 | 0.88 | 0.50 |  |  |  |  |  |
|  | G3f | 17.29 ± 3.65 | 0.84 | 1.00 |  |  |  |  |  |
|  | G4m | 13.84 ± 3.89 | 0.94 | 0.50 |  |  |  |  |  |
|  | G5f | 12.83 ± 2.60 | 0.92 | 0.75 |  |  |  |  |  |
|  | G6m | 15.35 ± 2.91 | 0.89 | 0.50 |  |  |  |  |  |
| cockatiel | C1m | 11.47 ± 4.63 | 0.69 | 0.50 | 0.09 | 0.802 | -0.42 | | 0.221 |
|  | C2m | 8.04 ± 1.29 | 0.74 | 0.50 |  |  |  |  |  |
|  | C3m | 11.68 ± 1.55 | 0.72 | 0.00 |  |  |  |  |  |
|  | C4m | 10.36 ± 1.89 | 0.81 | 0.50 |  |  |  |  |  |
|  | C5m | 9.15 ± 1.43 | 0.90 | 0.00 |  |  |  |  |  |
|  | C6f | 9.61 ± 1.46 | 0.66 | 0.50 |  |  |  |  |  |
|  | C7f | 11.09 ± 3.15 | 0.72 | 0.25 |  |  |  |  |  |
|  | C8f | 9.18 ± 1.61 | 0.83 | 0.00 |  |  |  |  |  |
|  | C9m | 11.74 ± 1.15 | 0.79 | 0.50 |  |  |  |  |  |
|  | C10m | 8.64 ± 1.67 | 0.68 | 0.25 |  |  |  |  |  |
| Task 5 | | | | | | | | | |
| galah | G1m | 5.15 ± 2.25 | 0.94 | 0.50 | 0.61 | 0.195 | 0.44 | | 0.387 |
|  | G2f | 11.28 ± 5.48 | 0.88 | 0.75 |  |  |  |  |  |
|  | G3f | 8.93 ± 5.22 | 0.96 | 1.00 |  |  |  |  |  |
|  | G4m | 4.76 ± 1.68 | 0.86 | 0.50 |  |  |  |  |  |
|  | G5f | 7.63 ± 3.47 | 0.90 | 1.00 |  |  |  |  |  |
|  | G6m | 6.76 ± 4.20 | 0.88 | 0.50 |  |  |  |  |  |
| cockatiel | C1m | 3.95 ± 1.36 | 0.85 | 0.00 | 0.11 | 0.750 | 0.33 | | 0.343 |
|  | C2m | 6.75 ± 2.24 | 0.93 | 0.50 |  |  |  |  |  |
|  | C3m | 4.68 ± 0.90 | 0.93 | 0.25 |  |  |  |  |  |
|  | C4m | 3.95 ± 1.30 | 0.92 | 0.50 |  |  |  |  |  |
|  | C5m | 4.67 ± 1.26 | 0.93 | 0.00 |  |  |  |  |  |
|  | C6f | 3.21 ± 0.94 | 0.92 | 0.50 |  |  |  |  |  |
|  | C7f | 4.15 ± 1.04 | 0.94 | 0.25 |  |  |  |  |  |
|  | C8f | 4.21 ± 0.87 | 0.94 | 0.00 |  |  |  |  |  |
|  | C9m | 3.81 ± 0.91 | 0.93 | 0.50 |  |  |  |  |  |
|  | C10m | 4.87 ± 1.10 | 0.94 | 0.50 |  |  |  |  |  |
| Task 6 | | | | | | | | | |
| galah | G1m | 8.61 ± 3.75 | 0.96 | 1.00 | -0.55 | 0.255 | 0.55 | | 0.255 |
|  | G2f | 13.97 ± 3.35 | 0.84 | 0.50 |  |  |  |  |  |
|  | G3f | 15.15 ± 2.22 | 0.92 | 0.50 |  |  |  |  |  |
|  | G4m | 12.58 ± 3.59 | 0.81 | 0.50 |  |  |  |  |  |
|  | G5f | 10.50 ± 2.84 | 0.90 | 0.50 |  |  |  |  |  |
|  | G6m | 14.56 ± 4.10 | 0.85 | 0.75 |  |  |  |  |  |
| cockatiel | C1m | 8.83 ± 3.01 | 0.79 | 0.50 | -0.61 | 0.058 | 0.44 | | 0.201 |
|  | C2m | 9.32 ± 3.67 | 0.86 | 0.50 |  |  |  |  |  |
|  | C3m | 9.91 ± 1.80 | 0.85 | 0.50 |  |  |  |  |  |
|  | C4m | 10.97 ± 1.57 | 0.74 | 0.25 |  |  |  |  |  |
|  | C5m | 9.67 ± 2.08 | 0.85 | 0.00 |  |  |  |  |  |
|  | C6f | 10.18 ± 2.85 | 0.90 | 0.50 |  |  |  |  |  |
|  | C7f | 9.65 ± 1.43 | 0.79 | 0.25 |  |  |  |  |  |
|  | C8f | 11.71 ± 2.65 | 0.74 | 0.00 |  |  |  |  |  |
|  | C9m | 8.09 ± 1.52 | 0.77 | 0.50 |  |  |  |  |  |
|  | C10m | 9.28 ± 2.09 | 0.90 | 0.50 |  |  |  |  |  |
| Task 7 | | | | | | | | | |
| galah | G1m | 6.98 ± 1.92 | 0.96 | 1.00 | -0.06 | 0.911 | 0.69 | | 0.130 |
|  | G2f | 6.98 ± 1.75 | 0.86 | 0.50 |  |  |  |  |  |
|  | G3f | 15.03 ± 2.46 | 0.85 | 0.75 |  |  |  |  |  |
|  | G4m | 6.22 ± 2.01 | 0.82 | 0.50 |  |  |  |  |  |
|  | G5f | 12.16 ± 2.42 | 0.91 | 0.50 |  |  |  |  |  |
|  | G6m | 12.45 ± 5.92 | 0.84 | 0.50 |  |  |  |  |  |
| cockatiel | C1m | 7.50 ± 2.88 | 0.89 | 0.50 | 0.08 | 0.832 | 0.31 | | 0.382 |
|  | C2m | 9.48 ± 2.54 | 0.90 | 0.50 |  |  |  |  |  |
|  | C3m | 6.75 ± 2.95 | 0.87 | 0.25 |  |  |  |  |  |
|  | C4m | 6.97 ± 2.25 | 1.00 | 0.25 |  |  |  |  |  |
|  | C5m | 8.13 ± 3.13 | 0.75 | 0.00 |  |  |  |  |  |
|  | C6f | 6.29 ± 2.20 | 0.93 | 0.50 |  |  |  |  |  |
|  | C7f | 6.47 ± 2.57 | 0.93 | 0.25 |  |  |  |  |  |
|  | C8f | 6.71 ± 2.72 | 0.93 | 0.50 |  |  |  |  |  |
|  | C9m | 8.05 ± 2.52 | 0.80 | 0.50 |  |  |  |  |  |
|  | C10m | 7.50 ± 2.89 | 0.88 | 0.50 |  |  |  |  |  |
